# Correlations between insulin-degrading enzyme and metabolic markers in patients diagnosed with type 2 diabetes, Alzheimer’s disease, and healthy controls: a comparative study

**DOI:** 10.1007/s12020-023-03603-4

**Published:** 2023-11-18

**Authors:** Helena Kullenberg, Jenny Rossen, Unn-Britt Johansson, Maria Hagströmer, Thomas Nyström, Maria Kumlin, Marie M. Svedberg

**Affiliations:** 1 Department of Health Promoting Science, Sophiahemmet University, Stockholm, Sweden; 2 Department of Clinical Science and Education, Karolinska Institutet, Södersjukhuset, Stockholm, Sweden; 3 Division of Physiotherapy, Department of Neurobiology, Care Sciences and Society, Karolinska Institutet, Stockholm, Sweden; 4 Academic Primary Health Care Centre, Region Stockholm, Stockholm, Sweden

**Keywords:** Alzheimer’s disease, Insulin-degrading enzyme, Metabolic disorder, Type 2 diabetes mellitus, Insulin resistance, Serum

## Abstract

**Purpose:**

This study aimed to explore correlations between insulin-degrading enzyme (IDE) and markers of metabolic function in a group of patients diagnosed with type 2 diabetes mellitus (T2DM) or Alzheimer’s disease (AD) and metabolically healthy volunteers.

**Method:**

We included 120 individuals (47 with T2DM, 9 with AD, and 64 healthy controls). Serum levels of IDE were measured with commercial kits for ELISA. Differences in IDE levels between groups were analyzed with non-parametric ANCOVA, and correlations were analyzed with Spearman’s rank correlations. We also investigated the influence of age, sex, and the use of insulin on the correlation using a non-parametric version of partial correlation.

**Results:**

Patients diagnosed with T2DM had higher IDE levels than patients diagnosed with AD and healthy controls after adjustment for age and sex. IDE was increasingly associated with body mass index (BMI), fasting blood glucose, C-peptide, hemoglobin A1c (HbA1c), insulin resistance, and triglycerides. In stratified analyses, we found a decreasing partial correlation between IDE and HbA1c in patients diagnosed with AD and a decreasing partial correlation between IDE and C-peptide in healthy controls. In patients diagnosed with T2DM, we found no partial correlations.

**Conclusion:**

These results indicate that IDE is essential in metabolic function and might reflect metabolic status, although it is not yet a biomarker that can be utilized in clinical practice. Further research on IDE in human blood may provide crucial insights into the full function of the enzyme.

## Introduction

Type 2 diabetes mellitus (T2DM) is a condition that affects the individual’s ability to regulate insulin and glucose levels. The diagnosis is associated with an altered lipid metabolism with high levels of free fatty acids and low protective adipokines, such as adiponectin [1]. This metabolic dysfunction leads to a significant risk of acute and long-term complications, of which cognitive impairment is one [2]. It has been increasingly recognized that individuals with T2DM are at a higher risk of developing Alzheimer’s disease (AD) [3]. The full pathophysiological link is yet to be revealed, but insulin resistance has been suggested as a common factor [4].

Insulin-degrading enzyme (IDE) has been proposed as a connecting biochemical marker between T2DM and AD [5]. It is a zinc-metalloprotease that has a vital role in insulin degradation and degrades beta-sheet-forming proteins, such as amyloid-beta (Aβ) in the brain and human islet amyloid polypeptide (hIAPP) in the pancreas [6]. In addition, the enzyme facilitates proper folding to prevent the aggregation of these proteins [7]. This has associated IDE with pancreatic beta-cell dysfunction in T2DM [8], neurodegeneration in AD 7, and glucose homeostasis [9].

It has previously been demonstrated by us and others that patients diagnosed with metabolic syndrome [10] or T2DM [11] have higher serum levels of IDE compared to metabolically healthy controls. At the same time, cognitive impairment has been associated with lower levels of IDE [12–14]. This has led to IDE being proposed as a biomarker in preventing and diagnosing AD [13, 14]. In addition, IDE can be analyzed in blood serum [9], making it an appealing possibility in a clinical setting. However, IDE is most likely influenced by several factors that must be considered, including age [15], ethnicity [10, 16], genetic variation [17, 18], and drug treatment, for instance, insulin [19], metformin [20], GLP-1 analogs [21], and ACE inhibitors [22]. Additionally, diet [23] and physical activity [24] have been suggested to affect the levels of IDE, perhaps even accounting for parts of the positive effects of lifestyle interventions in patients diagnosed with T2DM [5].

The correlations between IDE and various metabolic biomarkers, such as fasting blood glucose [12], insulin [10, 12], and triglycerides [10], could provide insight into the broader metabolic context in which IDE operates. This knowledge can help elucidate the intricate web of interactions within the metabolic system, which could be crucial for understanding the pathophysiology of T2DM and AD. Still, the evidence on IDE levels in human blood is limited, and the results of previous studies are contradictory, indicating that more knowledge is needed. Therefore, this study aimed to explore the correlation between IDE and biological markers important in metabolic function, such as age, weight, blood glucose, C-peptide, and lipids, in patients diagnosed with T2DM or AD compared to metabolically healthy volunteers.

## Materials and methods

### Study participants

Patients diagnosed with T2DM were selected from biobank no. 929 at Sophiahemmet Hospital. The selection of samples has been described earlier [11]; in brief, all subjects in the biobank diagnosed with T2DM were selected. These patients had been diagnosed with T2DM at healthcare centers in Sweden and were prescribed antidiabetic treatment following the national guidelines for glucose-lowering treatments of T2DM.

Patients diagnosed with AD were recruited at nursing homes. We invited all patients diagnosed with AD to participate. Exclusion criteria were a diagnosis of type 1 diabetes mellitus (T1DM), prediabetes, or T2DM.

Healthy volunteers aged 18 years or older were recruited at Sophiahemmet University and Sophiahemmet Health Center through written notices, brochures in waiting rooms or common areas, and information during lectures or medical appointments. To ensure that we recruited metabolically healthy individuals, we applied the following exclusion criteria: blood pressure >140/90 mm Hg, body mass index (BMI) > 30 kg/m^2^, fasting blood glucose >5.6 mmol/l, hemoglobin A1c (HbA1c) > 39 mmol/l, the diagnosis of T1DM, prediabetes, T2DM, or another medical diagnosis requiring regular drug treatment [25].

### Blood sample collection

Fasting blood samples were drawn from a decubital vein and collected in EDTA tubes (for whole blood analyses) and serum tubes. Serum tubes were left at room temperature to clot for 30 min and then centrifuged at 3000 rpm for 15 min. The supernatant was collected and frozen at −80 degrees until laboratory analyses. Whole blood analyses were conducted at the time of blood collection.

### Biochemical analysis of human serum samples

Enzyme-linked immunosorbent assays (ELISA) were used to measure IDE, C-peptide, and adiponectin levels in blood serum samples according to the manufacturers’ instructions. We used an automated plate washer (HydroWash, Tecan) for the required wash steps. Standard curves were fitted for all analyses using a 4 parametric logistic curve.

Serum IDE levels were measured using a Human Insulin-degrading enzyme ELISA Kit (AH Diagnostics, Solna, Sweden), with a detection limit of 0.037 ng/ml.

Serum C-peptide levels were measured by Human C-peptide ELISA (Merck, Stockholm, Sweden), with a detection limit of 0.05 ng/ml.

Serum adiponectin levels were measured by Adiponectin Human ELISA Kit (Invitrogen, Thermo Fisher Scientific, Stockholm, Sweden). The detection limit was 0.23 ng/ml.

For all analyses, the coefficients of variation (CV) were below 10%.

Lipids (triglycerides, high-density lipoproteins (HDL), and low-density lipoproteins (LDL)) were measured in serum with the point-of-care platform Afinion^TM^ 2 (Lipid panel, Abbott Rapid Diagnostics, Maidenhead, UK).

### Clinical data

For patients diagnosed with T2DM, information on age, sex, height, weight, fasting blood glucose, HbA1c, and drugs were available in the biobank database.

For participants recruited to this study (i.e., patients diagnosed with AD and healthy controls), information on age, sex, height, and weight was self-reported. For patients diagnosed with AD, the nursing home nurse helped to fill out the form with height and weight and provided a list of prescribed drugs. Fasting blood glucose and HbA1c were analyzed in whole blood using point-of-care platforms from HemoCue (HemoCue AB, Ängelholm, Sweden) and Afinion^TM^ 2 (Abbott Rapid Diagnostics, Maidenhead, UK), respectively.

BMI was calculated by dividing body weight in kilograms by the squared height in meters (kg/m^2^).

Insulin sensitivity was estimated using the computer calculator for hemostatic model assessment (HOMA2) [26]. In the calculator, we applied fasting blood glucose (mmol/l) and fasting C-peptide (ng/ml) to estimate the level of insulin resistance (HOMA2-IR). The computer model calculates a value for insulin resistance, where the magnitude of 1 is considered normal, and a higher value indicates a more pronounced insulin resistance [27].

### Statistical analysis

Following a test of normal distribution in data, we decided to use non-parametric analyses in this study since none of the variables were of normal distribution, and the sample size was relatively small.

Descriptive variables were presented as count (n) and percent (%) or median (m) and interquartile range (IQR) as suitable. Differences between groups were tested with Pearson’s chi-squared test or Kruskal-Wallis test by ranks. Since the distribution of age and sex differed between healthy controls, patients diagnosed with T2DM, and patients diagnosed with AD, a non-parametric variant of the one-way analysis of covariance (ANCOVA), also called Quade analysis, was used to analyze the difference between the groups in serum IDE levels. This statistical test allowed us to adjust for age and sex as covariates.

Bivariate correlations between measured variables were determined using Spearman’s rank correlation for unadjusted associations and a non-parametric partial correlation to adjust associations for age and sex. When analyzing all participants and patients diagnosed with T2DM, we adjusted for the use of insulin as a bivariate variable, user or non-user. Treatment with insulin has been suggested to upregulate IDE [19]. In addition, insulin treatment is generally connected to a lower endogen insulin production [28], which affects levels of C-peptide used to calculate HOMA2-IR in this study.

A *p*-value < 0.05 was considered statistically significant. The effect of multiple tests was corrected using the Bonferroni correction. All reported *p*-values were two-tailed. Statistical analyses were performed with IBM SPSS Statistics version 27.0 (IBM, NY, USA). Figures were produced in GraphPad Prism 9 (GraphPad Software Inc).

## Results

### Characteristics of the study sample

We included 120 individuals (47 patients diagnosed with T2DM, 9 with AD, and 64 healthy controls) (Table 1).

**Table 1:** Characteristics of the study sample (*n* = 120)

	T2DM	AD	Healthy controls	Sig.
Number of participants	47	9	64	
Sex, male, *n* (%)	38 (77.6)	2 (22.2)	20 (32.3)	<0.001
Age, years, m (IQR)	66.0 (57–71)	88.0 (82–89)	36.5 (26–52)	<0.001^a,b,c^
IDE, ng/ml, m (IQR)	30.75 (23.88–42.02)	11.93 (8.12–16.12)	7.60 (5.19–19.35)	<0.001^a,c^
HbA1c, mmol/mol, m (IQR)	55 (48–61)	37 (35–38)	32 (31–34)	0.000^a,c^
Fasting blood glucose, mmol/l, m (IQR)	8.2 (6.8–10.2)	4.3 (4.0–5.5)	5.0 (4.5–5.3)	<0.001^a,c^
C-peptide, ng/ml, m (IQR)	2.90 (2.00–3.60)	2.05 (1.38–3.07)	0.85 (0.59–1.02)	<0.001^a,b^
HOMA2-IR	2.36 (1.72–3.36)	1.43 (0.90–2.31)	0.69 (0.61–0.81)	<0.001^a,b^
BMI, kg/m^2^, m (IQR)	29.4 (27.5–32.6)	19.4 (18.9–22.66)	22.7 (20.8–24.0)	<0.001^a,c^
Adiponectin, ng/ml, m (IQR)	5.81 (3.52–9.28)	12.96 (7.67–18.78)	10.92 (7.04–17.34)	<0.001^a,c^
Triglycerides, mmol/l, m (IQR)	1.43 (0.90–2.00)	1.10 (0.77–1.58)	0.75 (0.64–0.96)	<0.001^a^
LDL, mmol/l, m (IQR)	2.5 (1.90–2.90)	3.42 (3.04–4.09)	2.70 (2.10–3.19)	0.013^c^
HDL, mmol/l, m (IQR)	1.09 (0.96–1.38)	1.49 (1.46–1.74)	1.46 (1.27–1.78)	<0.001^a,c^

Differences were analyzed with the Kruskal–Wallis test and corrected for multiple comparisons with Bonferroni. A *p*-value < 0.05 was considered significant

*AD* Alzheimer’s disease, *BMI* body mass index, *HbA1c* glycated hemoglobin A1c, *HDL* high-density lipoproteins, *HOMA2-IR* homeostatic model assessment of insulin resistance, *IDE* insulin-degrading enzyme, *IQR* interquartile range, *LDL* low-density lipoprotein, *m* median, *T2DM* type 2 diabetes mellitus

^a^Difference between T2DM and control

^b^Difference between AD and control

^c^Difference between T2DM and AD

Patients diagnosed with T2DM were younger than patients diagnosed with AD but older than the healthy controls. Further, they had statistically significantly higher BMI, fasting blood glucose, and HbA1c but lower adiponectin and HDL cholesterol levels than patients diagnosed with AD and healthy controls (Table 1).

There was no statistically significant difference between patients diagnosed with T2DM or AD regarding levels of C-peptide, HOMA2-IR, or triglycerides. Also, patients diagnosed with T2DM, or AD, had significantly higher C-peptide and HOMA2-IR than healthy controls. Patients diagnosed with AD had significantly higher LDL cholesterol levels than those with T2DM. Patients diagnosed with T2DM had higher levels of triglycerides than healthy controls (Table 1).

### IDE serum levels

Patients diagnosed with T2DM had higher serum IDE levels compared to patients diagnosed with AD (30.75 ng/ml vs. 11.93, *p* = 0.037) and healthy controls (30.75 ng/ml vs. 7.60, *p* = 0.001) (Table 1).

**Fig. 1:**
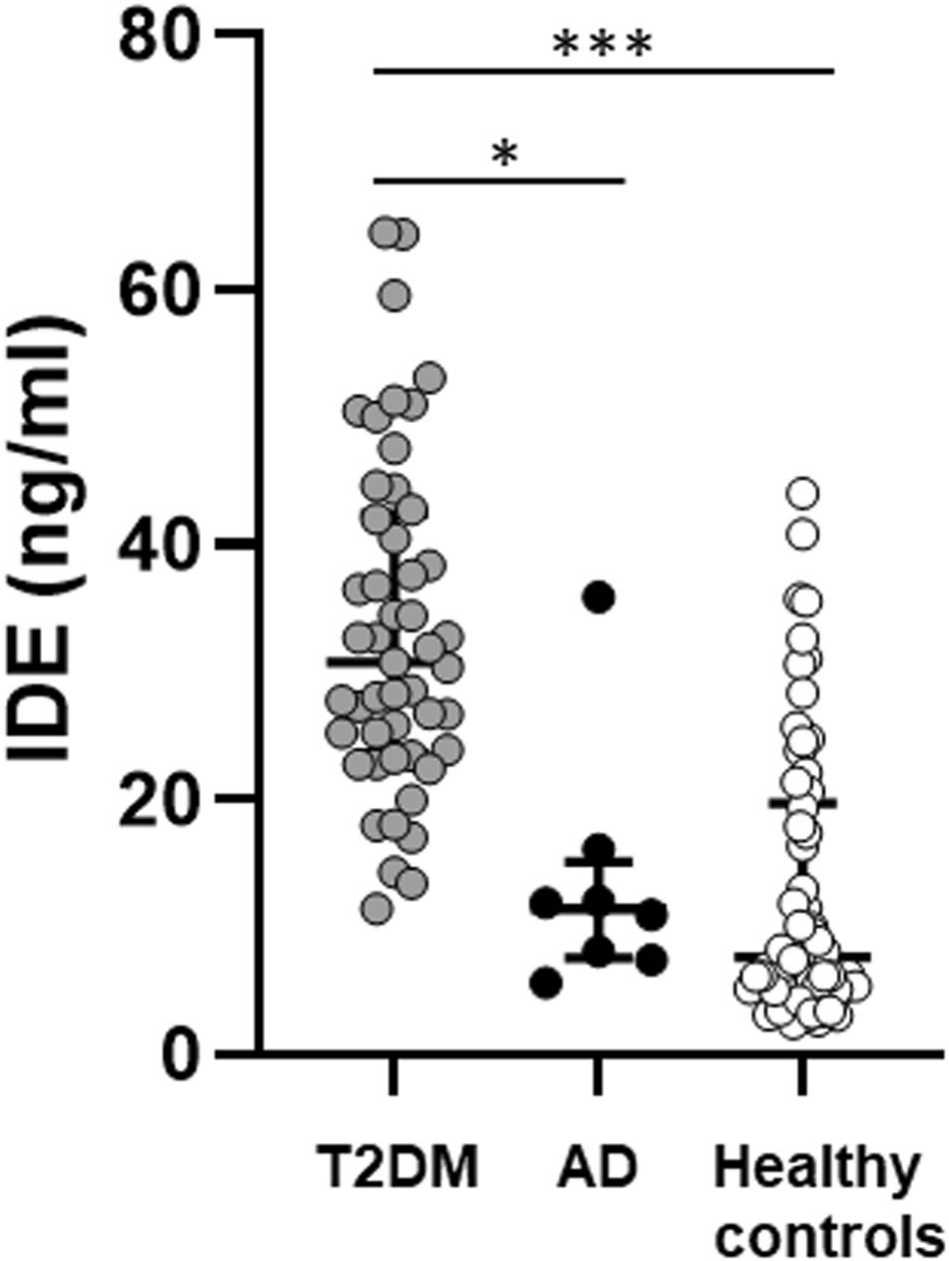
Serum levels of insulin-degrading enzyme (IDE) in patients diagnosed with type 2 diabetes mellitus (T2DM), Alzheimer’s disease (AD), and healthy controls. Differences were analyzed with a non-parametric ANCOVA and adjusted for age and sex. **p-value < 0.05 ***p-value < 0.001

The ANCOVA analysis demonstrated that the difference between patients diagnosed with T2DM and AD (*p* = 0.030) and patients diagnosed with T2DM and healthy controls (*p* < 0.001) remained after adjustment for age and sex (Fig. 1).

### Correlations between IDE and other biomarkers

Correlations were investigated for the total sample (all participants *n* = 120) and patients diagnosed with T2DM, AD, and healthy controls separately.

In the total sample, there was a highly significant correlation between IDE and age (*r* = 0.54, *p* < 0.001), sex (*r* = 0.40, *p* < 0.001), and the use of insulin (*r* = 0.37, *p* < 0.001) (Table 2). In addition, age, sex, and insulin use were significantly correlated with several metabolic variables, as shown in Table 3. However, when stratifying the groups, sex was not significantly associated with IDE levels in either group. Age was only significantly correlated in healthy controls (*r* = 0.27, *p* = 0.034). Insulin use was also not significantly associated with IDE levels when the T2DM group was solely included in the analysis (Table 2).

**Table 2:** Correlations between IDE and metabolic variables

	Total Sample (*n* = 120)				Patients diagnosed with T2DM (*n* = 47)				Patients diagnosed with AD (*n* = 9)				Healthy Controls (*n* = 64)			
	Unadjusted		Adjusted		Unadjusted		Adjusted		Unadjusted		Adjusted		Unadjusted		Adjusted	
	r	*p*	r	*p*	r	*p*	r	*p*	r	*p*	r	*p*	r	*p*	r	*p*
Sex, male	0.403	<0.001			−0.040	0.790			0.632	0.127			0.010	0.937		
Age	0.543	<0.001			0.162	0.282			0.291	0.527			0.274	0.034		
Insulin use	0.367	<0.001			0.006	0.966			-	-			-	-		
HbA1c	0.728	<0.001	0.473	<0.001	0.059	0.702	0.070	0.658	−0.916	0.001	−0.949	0.004	−0.028	0.853	−0.043	0.780
FBG	0.671	<0.001	0.497	<0.001	−0.004	0.981	−0.039	0.806	0.179	0.702	−0.016	0.976	0.079	0.547	0.076	0.572
C-peptide	0.693	<0.001	0.559	<0.001	0.242	0.088	0.270	0.061	−0.405	0.320	−0.590	0.217	−0.365	0.004	−0.341	0.008
HOMA2-IR	0.703	<0.001	0.565	<0.001	0.208	0.156	0.230	0.128	−0.405	0.320	−0.590	0.217	0.278	0.028	0.244	0.061
BMI	0.622	<0.001	0.448	<0.001	0.097	0.518	0.104	0.501	0.048	0.911	−0.004	0.993	0.083	0.583	0.083	0.590
Adiponectin	−0.345	<0.001	−0.125	0.220	0.002	0.991	−0.024	0.873	0.771	0.072	−0.274	0.765	−0.151	0.312	−0.045	0.771
Triglycerides	0.492	<0.001	0.329	0.001	0.083	0.585	0.127	0.419	−0.786	0.036	−0.647	0.238	0.065	0.623	−0.012	0.929
LDL	−0.052	0.612	−0.116	0.270	0.040	0.793	0.037	0.814	−0.357	0.432	−0.474	0.419	0.243	0.062	0.120	0.368
HDL	−0.459	<0.001	−0.291	0.005	−0.110	0.077	−0.131	0.402	0.357	0.432	−0.343	0.572	−0.171	0.190	−0.205	0.122

A *p*-value < 0.05 was considered significant

*AD* Alzheimer’s disease, *BMI b*ody mass index, *FBG* fasting blood glucose, *HbA1c g*lycated hemoglobin A1c, *HDL* high-density lipoproteins, *HOMA2-IR* homeostatic model assessment of insulin resistance, *IDE* insulin-degrading enzyme, *LDL* low-density lipoprotein, *T2DM* type 2 diabetes mellitus

Unadjusted (zero-order) correlations were analyzed with Spearman’s rank correlations test. Adjusted correlations were analyzed with non-parametric partial correlation and adjusted for age, sex, and use of insulin

**Table 3:** Non-parametric correlations between age, sex, and use of insulin and variables other than IDE—unadjusted correlations were analyzed with Spearman’s rank correlations test in the total sample (*n* = 120)

	Age		Sex, male		Insulin use	
	*r*	*p*	*r*	*p*	*r*	*p*
Age			0.196	0.032	0.242	0.008
Sex, male	0.196	0.032			0.311	<0.001
Insulin use	0.242	0.008	0.311	<0.001		
HbA1c	0.617	<0.001	0.445	<0.001	0.493	<0.001
FBG	0.380	<0.001	0.380	<0.001	0.370	<0.001
C-peptide	0.535	<0.001	0.305	<0.001	0.202	0.027
HOMA2-IR	0.572	<0.001	0.358	<0.001	0.180	0.049
BMI	0.330	<0.001	0.442	<0.001	0.445	<0.001
Adiponectin	−0.200	0.044	−0.466	<0.001	−0.157	0.116
Triglycerides	0.472	<0.001	0.196	0.034	0.195	0.036
LDL	0.206	0.028	−0.116	0.219	−0.155	0.100
HDL	−0.172	0.063	−0.512	<0.001	−0.314	<0.001

A *p*-value < 0.05 was considered significant

*BMI* body mass index, *FBG* fasting blood glucose, *HbA1c* glycated hemoglobin A1c, *HDL* high-density lipoproteins, *HOMA2-IR* homeostatic model assessment of insulin resistance, *IDE* insulin-degrading enzyme, *LDL* low-density lipoprotein

Further, the associations between IDE levels and the other metabolic variables were explored using partial correlation while controlling for age, sex, and the use of insulin (in all participants and the group diagnosed with T2DM). In all participants, there were moderate positive partial correlations between IDE and HbA1c (*r* = 0.47*, p* < 0.001), fasting blood glucose (*r* = 0.50*, p* < 0.001), C-peptide (*r* = 0.56*, p* < 0.001), HOMA2-IR (*r* = 0.57*, p* < 0.001), BMI (*r* = 0.45*, p* < 0.001), and triglycerides (*r* = 0.33*, p* = 0.001). In addition, there were moderate negative partial correlations between IDE and and HDL (*r* = −0.29*, p* = 0.005) (Table 2). An inspection of the zero-order correlation suggested that adjustment for age, sex, and insulin use substantially affected the strength of the relationship between these variables (Table 2).

**Fig. 2:**
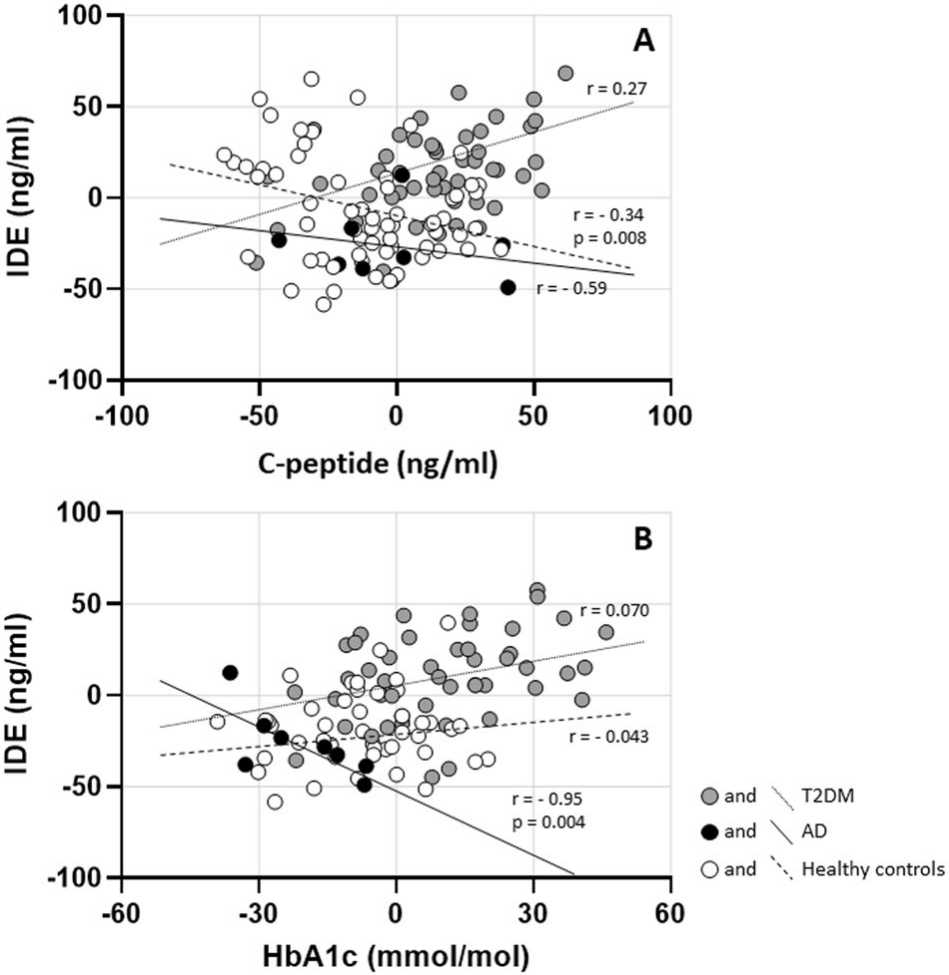
Partial correlation between insulin-degrading enzyme (IDE) and **A** C-peptide and **B** hemoglobin A1c (HbA1c)

When stratifying the groups, there was a moderate negative partial correlation between IDE and C-peptide (*r* = −0.34, *p* = 0.008) in the group of healthy controls (Fig. 2 and Table 2). The zero-order correlation (*r* = −0.37) demonstrated that adjustment for age and sex slightly decreased the strength of the relationship between these variables (Table 2). In the zero-order correlation, HOMA2-IR (*r* = 0.28, *p* = 0.028) and age (*r* = 0.27, *p* = 0.034) were significantly correlated with IDE in the control group but not after adjustment (Table 2).

In the group with AD, there was a strong negative partial correlation between IDE and HbA1c (*r* = −0.95, *p* = 0.004) (Fig. 2). The zero-order correlation (*r* = −0.92) suggested that the relation between IDE and HbA1c did increase following adjustment for age and sex. The zero-order correlation also showed a negative correlation between IDE and triglycerides (*r* = −0.786, *p* = 0.036) for patients diagnosed with AD. Still, adjustment for age and sex eliminated the relation between the variables (*r* = −0.65, *p* = 0.238) (Table 2).

In the groups with T2DM, we could not demonstrate any statistically significant correlations between IDE and measured metabolic variables (Table 2).

## Discussion

This study showed that patients diagnosed with T2DM had higher serum IDE levels than patients diagnosed with AD and metabolically healthy controls, respectively, even when considering age and sex. This confirms results from previous studies where metabolic dysfunction seems to be associated with increasing serum IDE levels [10, 11].

In addition, we demonstrated an increasing correlation between levels of IDE and fasting blood glucose, HbA1c, C-peptide, HOMA2-IR, BMI, and triglycerides in the whole sample. In contrast, adiponectin and HDL cholesterol showed a decreasing association. These findings align with previous studies, as Sofer and colleagues demonstrated that serum IDE levels were increasingly correlated with insulin, C-peptide, and triglycerides in patients diagnosed with metabolic syndrome [10]. However, we could also demonstrate a decreasing association between IDE levels and C-peptide in the group of metabolically healthy controls. This suggests that higher insulin levels in “normal” metabolism might reduce IDE levels. Likewise, we found a decreasing association between IDE levels and HbA1c in patients diagnosed with AD. These divergent results further suggest that IDE levels and perhaps IDE activity may differ depending on metabolic function.

In line with this, Sun and colleagues, who included patients diagnosed with T2DM with or without mild cognitive impairment, found that patients diagnosed with mild cognitive impairment had lower levels of IDE, which were negatively correlated with cognitive function, HOMA-IR, and fasting blood glucose [12]. In this study, we had access to a limited number of serum samples from patients diagnosed with AD, making it challenging to conclude the part of the results that included only these patients. Nevertheless, the statistical analyses demonstrated significant results for the decreasing association between IDE and HbA1c. Hence, the results showed that AD patients were similar to the healthy controls in many aspects regarding metabolic status. Interestingly, the patients diagnosed with AD had higher serum C-peptide levels and higher HOMA2-IR than healthy controls, suggesting that patients diagnosed with AD had insulin resistance at a level like those diagnosed with T2DM, even if they did not have elevated fasting blood glucose. This finding of insulin resistance in AD aligns with earlier studies [29]. A possible explanation could be that advanced age has been linked to insulin resistance [15, 30]. However, there was no difference in IDE levels between the healthy controls and AD patients, corresponding to this increased insulin resistance, as it appeared to be in the T2DM group. Although the group of patients diagnosed with AD was small and this limitation must be taken into consideration when interpreting the results, this finding could possibly be seen as a further indication that serum IDE levels do not merely respond to elevated insulin levels or peripheral insulin resistance.

Drug treatment in T2DM has been suggested to upregulate IDE levels [31, 32]. This could explain why patients diagnosed with T2DM did not show the same relationship between IDE levels and C-peptide as healthy controls. Metformin has even been proposed as a treatment for AD due to this increase in IDE [20]. In this study, we have only considered the use of insulin, as it might affect levels of C-peptide [28]. In a previous study, we investigated differences in serum IDE levels according to antidiabetic treatment, i.e., lifestyle treatment, peroral drug treatment, and insulin treatment. We found no difference in serum IDE levels between treatments [11]. However, here, we saw that the use of insulin was increasingly associated with serum IDE levels. Still, it did not significantly influence the relationship between IDE and other metabolic variables.

This study thus adds to the evidence that IDE can vary depending on an individual’s metabolic state. This, together with the drugs used in the treatment for T2DM, could explain why the enzyme behaves differently in patients with T2DM and healthy individuals. However, the significance of this in clinical practice remains to be investigated.

IDE activity is essential to maintain adequate insulin activity [33]. Concomitant hyperinsulinemia could drive high IDE levels [10]. However, it has been demonstrated that patients diagnosed with T2DM have decreased levels of IDE within the cytosol [34]. Consequently, more IDE must be synthesized to maintain adequate insulin degradation. This elevated level of IDE has been suggested to aggravate insulin resistance further [34]. Insulin resistance is connected to impairment in insulin signaling [35], and the compensatory overactivation of insulin signaling pathways may increase the secretion of Aβ [36] and the hyperphosphorylation of tau [37].

Even if there is no debate on whether Aβ is present in AD, the order of events has been discussed [38]. The amyloid cascade hypothesis stipulates that AD starts with the accumulation of proteins and that the plaques and neurofibrillary tangles cause inflammation, activated microglia, and subsequent neuronal death [38]. However, why the protein begins to accumulate has not yet been proven. The innate immune system and inflammation have been suggested as essential players [39]. It has been argued that age affects the immune system, causing an impaired adaptive immune response, increased production of pro-inflammatory cytokines, and a higher risk of autoimmune activity. This inflammaging could be why we see age-related diseases such as AD [40].

In a previous study, we found correlations between plasma levels of IDE and pro-inflammatory cytokines [41]. These analyses were done on post-mortem samples and must be confirmed in living patients. Still, inflammation has been suggested to induce oxidative stress, which is thought to impair activity in IDE [4]. Perhaps the higher serum IDE levels seen in T2DM in this study are due to a compensatory effect depending on lower enzyme activity caused by inflammation. This lower activity possibly increases the risk of developing AD if Aβ cannot be efficiently cleared. In addition, levels of IDE may have a more significant role in impaired glucose metabolism as IDE clears hIAPP in the pancreas, contributing to beta-cell function [42, 43]. Also, excessive insulin signaling, e.g., in insulin resistance, has been demonstrated to increase inflammation and promote aging further [44].

Nonetheless, one of the limitations of this study was the age differences between the groups being compared. There are conflicting results on whether age affects IDE, but IDE levels and activity are believed to decrease naturally over the lifespan. It has been argued that this could explain parts of the relationship observed between T2DM and AD [7, 15]. In this study, age was increasingly correlated with levels of IDE, but age was also increasingly associated with most of the metabolic markers measured. This multicollinearity made it impossible to build a reliable statistical model to examine age as an independent factor in relation to IDE level and adjust for other variables.

This speaks to the complexity of the relationship between metabolic function and neurodegeneration. Age is an independent risk factor for AD [45], but age also increases the risk of metabolic dysfunction [46]. As most metabolic markers seem to be nested with the levels of IDE, the enzyme may reflect an ongoing metabolic dysfunction that could affect the brain. Although, most studies investigating the expression of IDE have been done with brain tissue and CSF. Also, no studies have described serum IDE level changes over time in healthy individuals or patients diagnosed with T2DM or AD.

In clinical practice, IDE is currently not a marker that can be used. Further research is needed to understand its function and potential usefulness. In the meantime, however, the link between metabolic function and cognitive symptoms may be of great clinical interest. Correlations between IDE and other markers may further elucidate this relationship.

## Conclusion

Our findings demonstrate that T2DM patients exhibited elevated serum levels of IDE as compared to those with AD and healthy controls, even after adjusting for age and sex. Furthermore, we observed significant correlations between IDE serum levels and various metabolic markers, including HbA1c, C-peptide, HOMA2-IR, and lipids, across all samples. These results indicate that IDE is essential in metabolic function and might reflect metabolic status, although it is not yet a biomarker that can be utilized in clinical practice. Further research on IDE in human blood may provide crucial insights into the full function of the enzyme.

## Data Availability

The data presented in this study are available on request from the corresponding author. The data are not publicly available due to the ethical permit.
